# Group-Connected Impedance Network of RIS-Assisted Rate-Splitting Multiple Access in MU-MIMO Wireless Communication Systems

**DOI:** 10.3390/s23083934

**Published:** 2023-04-12

**Authors:** Min-A Kim, Seung-Geun Yoo, Hyoung-Do Kim, Kyeung-Ho Shin, Young-Hwan You, Hyoung-Kyu Song

**Affiliations:** 1 Department of Information and Communication Engineering, Sejong University, Seoul 05006, Republic of Korea; happy990927@sju.ac.kr (M.-A.K.); dbtmdrms96@sju.ac.kr (S.-G.Y.); gudeh8330@sju.ac.kr (H.-D.K.); shin980304@sju.ac.kr (K.-H.S.); 2 Department of Convergence Engineering for Intelligent Drone, Sejong University, Seoul 05006, Republic of Korea; yhyou@sejong.ac.kr; 3 Department of Computer Engineering, Sejong University, Seoul 05006, Republic of Korea

**Keywords:** reconfigurable intelligent surface, rate-splitting multiple access, mmWave, MIMO

## Abstract

The reconfigurable intelligent surface (RIS) and rate-splitting multiple access (RSMA) are considered as promising technologies for the beyond Fifth-Generation (B5G) and Sixth-Generation (6G) wireless systems by controlling the propagation environment, which attenuates the transmitted signal, and by managing the interference by splitting the user message into common and private messages. Because conventional RIS elements have each impedance connected to the ground, the sum-rate performance improvement of the RIS is limited. Therefore, the new RISs, which have impedance elements connected to each other, have been proposed recently. To be more adaptive to each channel, the optimization of the grouping of the RIS elements is required. Furthermore, since the solution of the optimal rate-splitting (RS) power-splitting ratio is complex, the value should be simply optimized to be more practical in the wireless system. In this paper, the grouping scheme of the RIS elements according to the user scheduling and the solution of the RS power-splitting ratio based on fractional programming (FP) are proposed. The simulation results showed that the proposed RIS-assisted RSMA system achieved a high sum-rate performance compared to the conventional RIS-assisted spatial-division multiple access (SDMA) system. Therefore, the proposed scheme can perform adaptively for the channel and has a flexible interference management. Furthermore, it can be a more suitable technique for B5G and 6G.

## 1. Introduction

Millimeter wave (mmWave) communication systems provide a high peak data rate and system capacity, but they result in restricted spectral efficiency (SE) because of severe signal attenuation [1,2]. Therefore, the beyond Fifth-Generation (B5G) and Sixth-Generation (6G) mobile communication systems require a more efficient use of the wireless resources and a more powerful means to manage the interference to provide services with a higher throughput and quality of service (QoS) [3]. The reconfigurable intelligent surface (RIS) and rate-splitting multiple access (RSMA) have attracted attention as promising technologies for B5G and 6G because of the improved SE [4,5,6,7,8].

The RIS, also called the intelligent reflecting surface (IRS), is composed of a large number of passive elements that can reflect the wireless signal independently in the desired direction. When the channel link of the base station (BS) and user equipment (UE) is blocked, the SE improvement is significant in RIS-based wireless communication. The RIS has various advantages, which are the ease of fabrication and deployment, low energy consumption, and compatibility with the techniques of conventional wireless networks. With these advantages, the applications of the RIS in diverse wireless communication networks have dramatically increased [9,10]. In recent years, a new RIS structure has been proposed, whereby each RIS element can be controlled through an interconnected impedance network, unlike the existing structure controlled by the impedance connected to the ground [11,12,13,14,15]. In [11,15], a reconfigurable impedance network matrix that maximizes the received power was found for single-user single-input single-output (SU-SISO) systems. A matrix with a continuous value was directly optimized by using the quasi-Newton method in [11], but a matrix with a discrete value was optimized by designing the discrete reactance codebook in [15]. In [12,13,14], the matrix that maximizes the sum-rate was optimized for multi-user multiple-input multiple-output (MU-MIMO) systems. An RIS architecture that supports reflecting and transmitting mode was considered in [12,14], and a multi-sector RIS structure with multiple RIS faces was proposed in [13].

RSMA can partially decode the interference and treat it as noise by splitting the user message into common and private messages. The split messages are independently encoded into common and private streams. The common streams are shared by multiple users, and the private streams are for each user. Due to the flexibility of the interference management, RSMA outperforms other multiple access techniques [3]. This technique has robustness to imperfect channel state information (CSI) [16,17]. Therefore, RSMA can mitigate the disadvantages of the RIS, which has inaccurate CSI for the RIS-aided wireless channel because of the passive elements of the RIS.

### 1.1. Contribution

In [14], a practical iterative algorithm was proposed to optimize the grouping scheme that maximizes the sum-rate, and in [15], a grouping method that maximizes the cosine similarity of the channel between the BS and RIS and the channel between the RIS and UE was proposed. In contrast, this paper proposes RSMA transmission-based RIS impedance network grouping in an MU-MIMO system through user scheduling. Furthermore, a simple scheme for obtaining the complicated optimal RS power-splitting ratio [18] is proposed. The main contributions of the paper are summarized as follows:

- First, the impedance network grouping scheme of the RIS is proposed for the MU-MIMO system. We obtained the existing RIS scattering matrix that maximizes the sum-rate. Then, the user scheduling was determined according to the effective channel gain of each user. To maximize the sum-rate, RIS elements having a high channel gain were grouped in the order of user scheduling.
- Second, to reduce the computational complexity, the scattering matrix of the impedance network grouped by the proposed method was successively optimized for each group using the quasi-Newton method. Therefore, the computation of the quasi-Newton method is effectively reduced.
- Finally, zero-forcing (ZF) precoding was used for the private data to cancel inter-user interference, and singular-value decomposition (SVD) precoding was considered for the common data. To find the optimal RS power-splitting ratio that maximizes the sum-rate, the problem derived by fractional programming was differentiated.

### 1.2. Organization and Notation

The rest of this paper is organized as follows. Section 2 describes the system model with the RIS by which each impedance is interconnected. In Section 3, the proposed scheme for impedance network grouping and the solution of the RS power-splitting ratio is provided. In Section 4, the simulation results of proposed system are presented. Finally, the conclusion of this paper is presented in Section 5.

In this paper, vectors and matrices are denoted with bold lowercase and bold uppercase letters, respectively. $A^{T},A^{H},A^{-1},{\left[\begin{array}{c} A \end{array}\right]}_{i,j}$, and $∥\begin{array}{c} A \end{array}∥$ refer to the transpose, conjugate transpose, inverse, (i,j)-th element, and spectral norm of matrix A, respectively. R and C denote the real and complex number sets, respectively. $j=\sqrt{-1}$ denotes the imaginary unit. 0 and I denote an all-zero matrix and an identity matrix, respectively, with appropriate dimensions. $\mathrm{CN}\left(\begin{array}{c} 0,I \end{array}\right)$ denotes the distribution of a circularly symmetric complex Gaussian random vector with mean vector 0 and covariance matrix I, and ∼ stands for “distributed as”. $\mathrm{diag}\left(\begin{array}{c} A_{1},\cdots ,A_{N} \end{array}\right)$ refers to a block diagonal matrix with blocks with $A_{1},\cdots ,A_{N}$.

## 2. System Model

**Figure 1:**
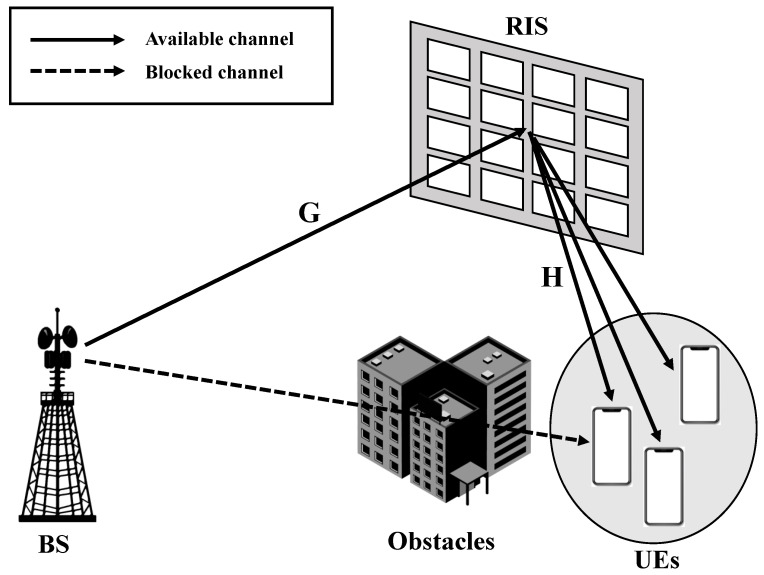
RIS-assisted MU-MIMO downlink system.

Figure 1 shows an RIS-assisted MU-MIMO downlink system. The considered system includes a BS equipped with $N_{T}$ transmit antennas, an RIS with $N_{I}$ elements, and $N_{K}$ UEs equipped single receive antennas. The scattering matrix of the $N_{I}$ RIS elements connected to the reconfigurable impedance network is represented as $\Phi \in C^{N_{I}\times N_{I}}$. The reconfigurable impedance network consists of passive elements that can be adjusted to the system by reflecting the impinging signal. According to network theory [19], the scattering matrix Φ can be expressed as follows:

(1) $$\Phi ={\left(Z_{I}+Z_{0}I\right)}^{-1}\left(Z_{I}-Z_{0}I\right),$$

where $Z_{I}$ refers to the impedance matrix and $Z_{0}$ refers to the characteristic impedance and is usually set as 50Ω. To maximize the power reflected by the RIS, $Z_{I}$ was assumed to be purely reactive as follows: $Z_{I}=jX_{I}$, where $X_{I}\in R^{N_{I}\times N_{I}}$ is the reactance matrix.

It was assumed that the direct channels between the BS and UEs are blocked by obstacles. The channels from the BS to the RIS and from the RIS to UEs are, respectively, denoted as $G\in C^{N_{I}\times N_{T}}$ and $H={\left[h_{1},h_{2},\cdots ,h_{N_{K}}\right]}^{T}\in C^{N_{K}\times N_{I}}$, where $h_{k}\in C^{1\times N_{I}}\left(i=1,2,\cdots ,N_{K}\right)$ refers to the channel from the RIS to the *k*-th UE. In this paper, the 3rd Generation Partnership Project (3GPP) TR 38.900 3-dimensional (3D) spatial channel model (SCM) was employed to apply the mmWave wireless communication [20,21]. In addition, the presence of a line of sight (LOS) component in each channel was considered. The cascaded channel between the RIS and UEs can be written as $H_{eff}=H\Phi G$. We denote the transmit signal as $x\in C^{N_{T}\times 1}$ with the transmit power constraint $E\left\{x^{H}x\right\}\leq P_{T}$. Then, the received signal at the *k*-th UE is represented as follows:

(2) $$y_{k}=h_{k}\Phi Gx+n_{k},$$

where $n_{k}∼\mathrm{CN}\left(0,\sigma_{k}^{2}\right)$ is the additive white Gaussian noise (AWGN).

### 2.1. Reconfigurable Impedance Network Connected by Group

The group-connected reconfigurable impedance network architecture is proposed to provide improved performance with low complexity [11]. The group-connected impedance network means that the impedance network is connected by the group. In this architecture, the $N_{I}$ RIS elements are grouped into $N_{G}$ groups. The elements in a group are connected by the impedance to all other elements in the group to form an “impedance network”. The scattering matrix of this architecture can be written as a block diagonal matrix in contrast to a conventional RIS which can usually be represented as a diagonal matrix. The reactance matrix of this architecture is a block diagonal matrix that satisfies the symmetric condition.The impedance networks have impedances connected to each other in the grouped elements, as well as grounded impedances for each element. The reactance matrix is expressed as a block-diagonal matrix that satisfies the symmetric condition; therefore, the RIS elements can be in different states. Then, the scattering matrix of this architecture can be written as a block diagonal matrix, in contrast to a conventional RIS, which can usually be represented as a diagonal matrix. Therefore, the reactance matrix and scattering matrix are given as follows:

(3) $$X_{I}=\mathrm{diag}\left(X_{I,1},X_{I,2},\cdots ,X_{I,N_{G}}\right),X_{I,g}=X_{I,g}^{T}\left(g=1,2,\cdots ,N_{G}\right),$$

(4) $$\Phi =\mathrm{diag}\left(\Phi_{1},\Phi_{2},\cdots ,\Phi_{N_{G}}\right),\Phi_{g}=\Phi_{g}^{T},\Phi_{g}^{H}\Phi_{g}=I\left(g=1,2,\cdots ,N_{G}\right).$$

According to [19], the scattering matrix should satisfy the unitary condition in addition to the symmetric condition.

### 2.2. Transmit Signal for RSMA

The RSMA scheme has various strategies that split user messages into common and private streams [3]. In this paper, the one-layer RS, which is the simplest and most-practical RSMA scheme, was assumed. At the BS, each user message is split by the message splitter, and the common messages of each user are combined into one common message by the message combiner. Then, the common message is encoded into a common stream $s_{c}$ by a codebook, which decodes the common message for all users. However, the private message is independently encoded into private streams $s_{1},s_{2},\cdots ,s_{N_{K}}$. The encoded streams $s={\left[s_{c},s_{1}.s_{2},\cdots ,s_{N_{K}}\right]}^{T}\in C^{(N_{K}+1)\times 1}$ were assumed to have zero mean and unit variance. Then, the streams are precoded by the linear precoder, which is represented as $P=\left[p_{c},p_{1},p_{2},\cdots ,p_{N_{K}}\right]\in C^{N_{T}\times \left(N_{K}+1\right)}$. Thus, the transmit signal is written as follows:

(5) $$x=\sqrt{P_{c}}p_{c}s_{c}+\sum_{k=1}^{N_{K}}\sqrt{P_{p}}p_{k}s_{k},$$

where $P_{c}=\left(1-\alpha \right)P_{T}$, $P_{p}=\frac{\alpha P_{T}}{N_{K}}$ is the power allocated to each stream, and α∈(0,1] is the RS power-splitting ratio. In this paper, the SVD [22,23] and ZF [24,25,26] precoding schemes was adopted at $p_{c}$ and $p_{k}$, respectively. The SVD precoder is given by the dominant left singular vector of the cascaded channel as follows:

(6) $$\begin{array}{c} H_{eff}^{T}=U\sum V^{H},\\ p_{c}={\left({\left[U\right]}_{1,1},{\left[U\right]}_{2,1},\cdots ,{\left[U\right]}_{N_{T},1}\right)}^{T}. \end{array}$$

Furthermore, the ZF precoder used in the private stream precoder $P_{p}=\left[p_{1},p_{2},\cdots p_{N_{K}}\right]$ is given by the pseudo-inverse of the cascaded channel as follows:

(7) $$\begin{array}{c} W=H_{eff}^{H}{\left(H_{eff}H_{eff}^{H}\right)}^{-1},\\ P_{p}=\frac{W}{∥\begin{array}{c} W \end{array}∥}. \end{array}$$

Hence, the received signal at the *k*-th UE can be rewritten as follows:

(8) $$y_{k}=\sqrt{P_{c}}h_{k}\Phi Gp_{c}s_{c}+\sqrt{P_{p}}h_{k}\Phi Gp_{k}s_{k}+\sum_{j\neq k}^{N_{K}}\sqrt{P_{p}}h_{k}\Phi Gp_{j}s_{j}+n_{k}.$$

## 3. Scattering Matrix with Grouping Scheme and Power Allocation

In this section, we derive the sum-rate problem for the RIS-assisted RSMA in the MU-MIMO wireless system and address the problem of grouping the RIS elements and power allocation to maximize the sum-rate performance. First, the common stream is decoded considering other private streams as interference. Then, each UE performs successive interference cancellation (SIC) to eliminate the common stream and decodes each private stream. The signal-to-interference-plus-noise ratio (SINR) of the common stream and private stream of the *k*-th UE is given as follows:

(9) $$\gamma_{c,k}=\frac{P_{c}{\left|h_{k}\Phi Gp_{c}\right|}^{2}}{\sum_{j=1}^{N_{K}}P_{p}{\left|h_{k}\Phi Gp_{j}\right|}^{2}+\sigma_{k}^{2}},\gamma_{p,k}=\frac{P_{p}{\left|h_{k}\Phi Gp_{k}\right|}^{2}}{\sum_{j\neq 1}^{N_{K}}P_{p}{\left|h_{k}\Phi Gp_{j}\right|}^{2}+\sigma_{k}^{2}}.$$

To ensure that common stream is decoded by all UEs, the SINR of the common stream is represented as $\gamma_{c}=\min\gamma_{c,k}$. Then, the sum-rate is given as follows:

(10) $$R=\log_{2}\left(1+\gamma_{c}\right)+\sum_{k=1}^{N_{K}}\log_{2}\left(1+\gamma_{p,k}\right).$$

In this paper, the scattering matrix with the grouping of the RIS elements and RS power ratio was optimized to maximize the sum-rate. The sum-rate problem for RIS-assisted RSMA in MU-MIMO can be formulated as follows:

(11) $$\;\max_{\Phi ,\alpha }R$$

(12) $$\;s.t.\Phi =\mathrm{diag}(\Phi_{1},\Phi_{2},\cdots ,\Phi_{N_{G}}),$$

(13) $$\;\Phi_{g}={\left(jX_{I,g}+Z_{0}I\right)}^{-1}\left(jX_{I,g}-Z_{0}I\right),\forall g,$$

(14) $$\;X_{I,g}=X_{I,g}^{T},\forall g.$$

To solve Equation (11), the alternative optimization algorithm is proposed. First, the scattering matrix optimization is performed, and then, the RS power ratio problem is solved.

### 3.1. Scattering Matrix with Grouping Scheme of the RIS Elements

When the RS power ratio is given, Equation (11) can be directly optimized by the quasi-Newton method [27] because the problem is an unconstrained optimization problem. However, the computational complexity increases as the number of RIS elements increases. In this paper, the problem was optimized sequentially for each group to reduce the complexity. Then, the sub-problem is given as follows:

(15) $$\begin{array}{c} \max_{\Phi_{g}}R_{g}\\ \;\;\;s.t.(12)-(14), \end{array}$$

where $R_{g}$ is the sum-rate of the effective channel corresponding to the group. The channel can be expressed as ${\left[h_{k}\right]}_{1,S_{g}}\Phi_{g}{\left[G\right]}_{S_{g},1:N_{T}}$.

**Figure 2:**
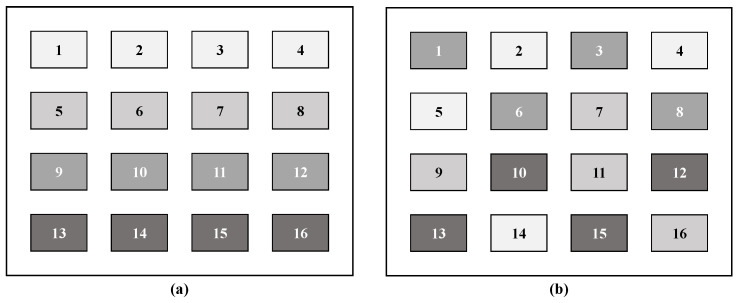
(**a**) General grouping; (**b**) proposed grouping; the numbers in the figure indicate the index of RIS elements.

Algorithm 1 specifies the overall optimization of Equation (11) with the grouping of RIS elements scheme. To obtain the grouping strategy, the scattering matrix needs to be initialized. In this paper, the conventional RIS scattering matrix that maximizes the channel gain was used as the initial scattering matrix. After the initialization of the scattering matrix, user scheduling is performed in descending order of the effective channel gain. By performing user scheduling, the scattering matrix can be more adaptive to each UE channel. Then, each RIS element is grouped by allocating the RIS element having the largest channel gain for each UE according to the user scheduling. In this scheme, the number of groups should be greater than the number of UEs in the system. Figure 2 shows the grouping strategy obtained through the proposed grouping scheme and the grouping between adjacent RIS elements represented by general grouping. In Figure 2, the scattering matrix index is based on the index of the figure, and the RIS elements in the same group appear in the same color, e.g., the 1st, 3rd, 6th, and 8th elements of Figure 2b are grouped into the same group.
**Algorithm 1** Optimization scattering matrix.**Input:** H, G, P, $N_{I}$, $N_{G}$, $N_{K}$, $P_{T}$, $\sigma_{k^{2}}$, ∀k**Output:**User scheduling: $\tilde{K}$Grouping strategy: $S_{1},S_{2},\cdots ,S_{N_{G}}$Scattering matrix: Φ  1:Initialize Φ  2:**for** $k=1:N_{K}$ **do**  3:      $\tilde{K}\left[k\right]=\max_{k}{∥h_{k}\Phi G∥}^{2}$  4:      ${∥h_{\tilde{K}\left[k\right]}\Phi G∥}^{2}=0$  5:**end for**  6:**for** $g=1:N_{G}$ **do**  7:      $\hat{g}=g\;mod\;N_{K}$  8:      **for** $i=1:N_{I}/N_{G}$ **do**  9:           $\hat{m}=\underset{m}{max}{∥h_{\tilde{K}\left[\hat{g}\right]}\Phi_{m,m}G∥}^{2}$   10:           $S_{g}=S_{g}\cup \left\{\hat{m}\right\}$   11:      **end for**   12:      $\Phi_{g}\leftarrow$ Solving Equation (15) by quasi-Newton method   13:**end for**

### 3.2. Power Allocation of the RS

Finally, the RS power ratio should be optimized, but the objective function is a nonconvex function. To optimize the RS power ratio simply, the sub-problem based on fractional programming (FP) by introducing four auxiliary variables is considered as follows:

(16) $$\begin{array}{cc} \max_{\alpha ,\nu_{c},\mu_{c},\nu_{k},\mu_{k},\forall k} & \log_{2}\left(1+\nu_{c}\right)-\nu_{c}+2\mu_{c}\sqrt{\left(1+\nu_{c}\right)P_{c}{\left|h_{\hat{k}}\Phi Gp_{c}\right|}^{2}}-\mu_{c}^{2}P_{c}{\left|h_{\hat{k}}\Phi Gp_{c}\right|}^{2}\\ & -\sum_{k=1}^{N_{K}}\mu_{c}^{2}P_{p}{\left|h_{\hat{k}}\Phi Gp_{k}\right|}^{2}+\mu_{c}^{2}\sigma_{\hat{k}}^{2}+\sum_{k=1}^{N_{K}}\{\log_{2}\left(1+\nu_{k}\right)-\nu_{k}\\ & +2\mu_{k}\sqrt{\left(1+\nu_{k}\right)P_{p}{\left|h_{k}\Phi Gp_{k}\right|}^{2}}-\sum_{j\neq k}\mu_{k}^{2}P_{p}{\left|h_{k}\Phi Gp_{j}\right|}^{2}+\mu_{k}^{2}\sigma_{k}^{2}\}\\ \; & \;s.t.(12)-(14), \end{array}$$

where $\hat{k}=arg\min_{k}\gamma_{c,k}$ is the UE that has the minimum SINR of the common stream. Then, the problem can be optimized by updating one variable while fixing the others. The optimal variables can be determined by setting ∂(16)/∂x to 0, where $x=\{\nu_{c},\mu_{c},\nu_{k},\mu_{k},\alpha \},\forall k$, as follows:

(17) $$\begin{array}{cc} \; & \nu_{c}=\frac{P_{c}{\left|h_{\hat{k}}\Phi Gp_{c}\right|}^{2}}{\sum_{k=1}^{N_{K}}P_{p}{\left|h_{\hat{k}}\Phi Gp_{k}\right|}^{2}+\sigma_{\hat{k}}^{2}},\mu_{c}=\frac{\sqrt{\left(1+\nu_{c}\right)P_{c}{\left|h_{\hat{k}}\Phi Gp_{c}\right|}^{2}}}{P_{c}{\left|h_{\hat{k}}\Phi Gp_{c}\right|}^{2}+\sum_{k=1}^{N_{K}}P_{p}{\left|h_{\hat{k}}\Phi Gp_{k}\right|}^{2}+\sigma_{\hat{k}}^{2}}, \end{array}$$

(18) $$\begin{array}{cc} \; & \nu_{k}=\frac{P_{p}{\left|h_{k}\Phi Gp_{k}\right|}^{2}}{\sum_{j\neq k}P_{p}{\left|h_{k}\Phi Gp_{j}\right|}^{2}+\sigma_{k}^{2}},\mu_{k}=\frac{\sqrt{\left(1+\nu_{k}\right)P_{p}{\left|h_{k}\Phi Gp_{k}\right|}^{2}}}{\sum_{j\neq k}P_{p}{\left|h_{k}\Phi Gp_{j}\right|}^{2}+\sigma_{k}^{2}},\forall k, \end{array}$$

(19) $$\begin{array}{cc} \; & \alpha =\hat{\alpha }, \end{array}$$

(20) $$\begin{array}{c} -\frac{\sqrt{\left(1+\nu_{c}\right)\mu_{c}^{2}P_{T}{\left|h_{\hat{k}}\Phi Gp_{c}\right|}^{2}}}{\left(1.5-\alpha \right)/\sqrt{2}}+\frac{P_{T}\sum_{k=1}^{N_{K}}\mu_{k}\sqrt{\left(1+\nu_{k}\right){\left|h_{k}\Phi Gp_{k}\right|}^{2}}}{\left(\alpha -0.5\right)/\sqrt{2}}+\mu_{c}^{2}P_{T}{\left|h_{\hat{k}}\Phi Gp_{c}\right|}^{2}\\ -\frac{P_{T}}{N_{K}}\sum_{k=1}^{N_{K}}\mu_{c}^{2}{\left|h_{\hat{k}}\Phi Gp_{k}\right|}^{2}-P_{T}\sum_{k=1}^{N_{K}}\sum_{j\neq k}\mu_{k}^{2}{\left|h_{k}\Phi Gp_{j}\right|}^{2}+\mu_{k}^{2}\sigma_{k}^{2}=0, \end{array}$$

where $\hat{a}$ is the solution of Equation (20), which is provided in Appendix A in detail. Equation (20) can be solved by a simple quadratic equation. The overall optimization is described in the following Algorithm 2.

In Algorithm 2, instead of calculating the initial value of α according to the channel information, the calculation of the algorithm is reduced by selecting the median value in the range of possible numbers. Through the simulation results in the next section, it was confirmed that the sum-rate performance can be optimized without additional calculation.
**Algorithm 2** Optimization RS power-splitting ratio.**Input:** H, G, Φ, P, $P_{T}$, $\sigma_{k^{2}}$, ∀k**Output:**RS power-splitting ratio: α
  1:Initialize α  2:**while** The value of the sum-rate converges. **do**  3:    Update $\nu_{c}$ by (17).  4:    Update $\mu_{c}$ by (17).  5:    Update $\nu_{k},$∀k by (18).  6:    Update $\mu_{k},$∀k by (18).  7:    Update α by (19).  8:**end while**

## 4. Simulation Results

### 4.1. Simulation Settings

In this section, the simulation results performed with Matlab for the proposed RIS-assisted RSMA scheme are presented. The 3D SCM channel was considered for the system. The UEs with a height of 1.5 m were randomly located at a distance of 5 m (50 m, 5 m). The positions of the BS and the RIS were, respectively, (0, 0, 25 m) and (50 m, 5 m, 5 m). The distance-based path-loss model was considered as $PL\left(d\right)=L_{0}d^{-\beta }$, where $L_{0}$ is the reference path loss at a distance of 1 m, d is the distance between the end nodes, and β is the path-loss exponent. The ratio coefficient between the LoS power and non-LoS power was set to 3 dB, and the inter-antenna and inter-RIS element spacing were set as half a wavelength. Table 1 shows the other simulation parameters.

### 4.2. Convergence Analysis

**Figure 3:**
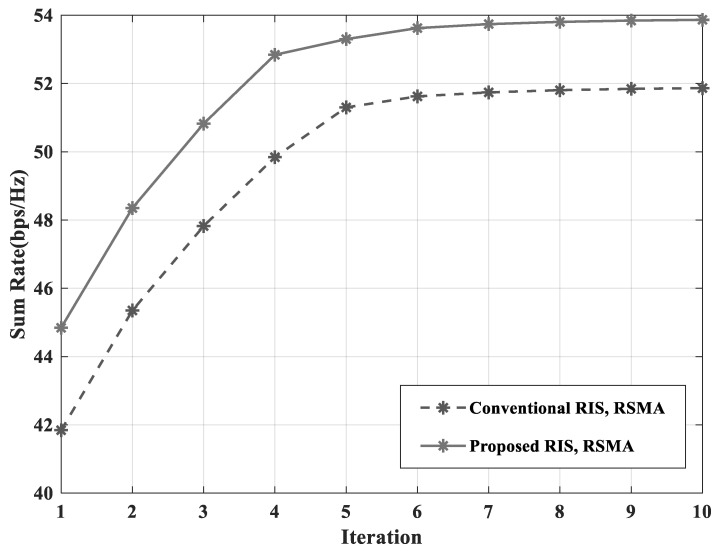
Sum-rate versus iteration with $N_{G}=4$ and $N_{I}=16$.

In this paper, to evaluate the performance of proposed scheme, the sum-rate in (10) was adopted. Figure 3 shows the sum-rate according to the number of iterations in Algorithm 2. One channel realization was considered in this simulation. The optimization algorithm of the RS power-splitting ratio can converge in 10 iterations. Moreover, it can be seen that the sum-rate performances were optimized with a gain of about 10 by the algorithm.

### 4.3. Sum-Rate Analysis

**Figure 4:**
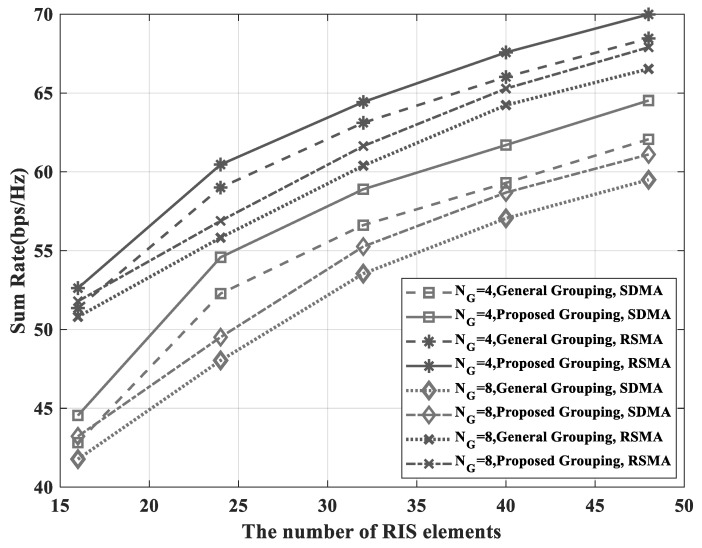
Sum-rate versus the number of RIS elements.

Figure 4 shows the sum-rate performance according to the number of RIS elements. It is shown that the RSMA transmit scheme outperformed the spatial division multiple access (SDMA) transmit scheme. With the proposed grouping scheme, the performance of the RSMA scheme had a gain of 7.7% and 9.8%, respectively, over the SDMA performance, when $N_{I}$ was 48. Additionally, the proposed grouping achieved higher performance than the general grouping. This means that the proposed grouping had the proper grouping strategy. For $N_{G}=4$, better performance was also seen; in other words, fewer groups resulted in higher sum-rate performance. However, fewer groups required more impedance value computation. Therefore, the reduction of the number of groups increased the complexity of the system. By using the RSMA scheme, the amount of RIS computation can be reduced and the sum-rate performance can also be increased. Moreover, the sum-rate of the proposed grouping scheme with $N_{G}=8$ achieved the sum-rate of the general grouping with $N_{G}=4$ in both the RSMA scheme and the SDMA scheme. It was seen that it had a low computational complexity and improved the performance effectively.

**Figure 5:**
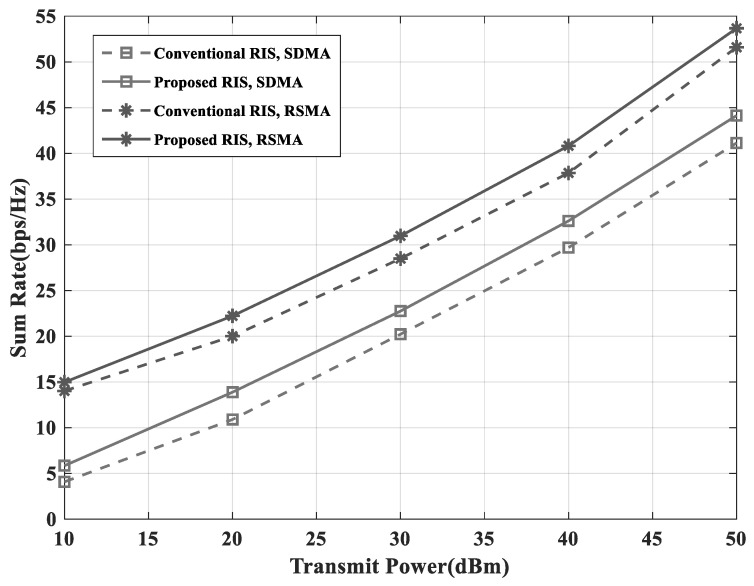
Sum-rate versus transmit power with $N_{G}=4$ and $N_{I}=16$.

**Figure 6:**
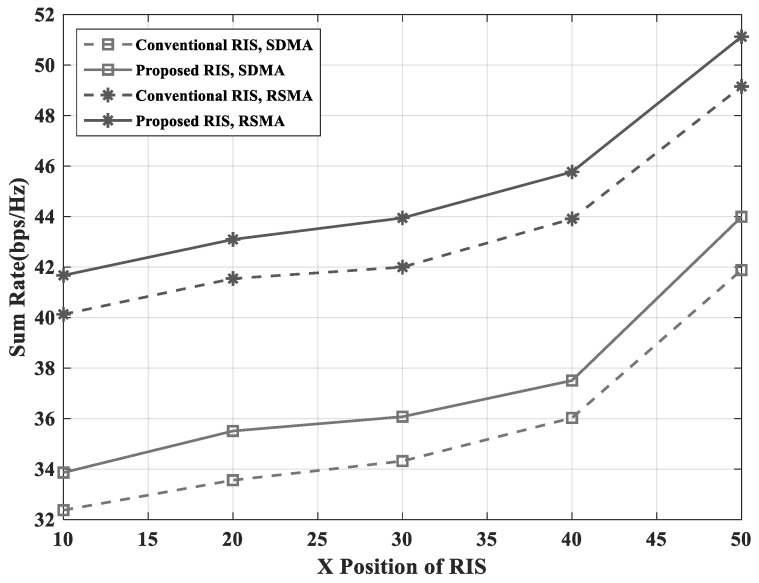
Sum-rate versus X position of the RIS with $N_{G}=4$ and $N_{I}=16$.

Figure 5 and Figure 6 show that the proposed RIS-assisted RSMA scheme had a higher sum-rate performance than the conventional RIS-assisted SDMA, because the proposed grouping was more adaptive and the RSMA scheme was more flexible in managing the interference. In the simulations, Algorithms 1 and 2 were applied as the proposed grouping scheme and the optimization of RS power-splitting ratio. Furthermore, the number of groups in the simulation was a number that could be divided by the number of elements. Since the number of users was set to 3, the number of groups should be greater than 3 to apply Algorithm 1. Therefore, in this paper, the number of groups was set to 4 and 8. Figure 5 shows the sum-rate performance according to the transmit power of the BS. The RSMA scheme achieved almost the same performance with about 10dBm lower power than the SDMA scheme. Furthermore, the proposed RIS had more improved performance than the conventional RIS. Therefore, it is demonstrated that proposed RIS-assisted RSMA scheme that had a similar performance with a low transmit power was more energy efficient than the conventional RIS-assisted SDMA scheme. In Figure 6, it is shown that the sum-rate performance improved when the position of the RIS was close to the UEs. However the RSMA scheme ensured a higher sum-rate than the SDMA scheme, even if the RIS was far away from the UEs.

**Figure 7:**
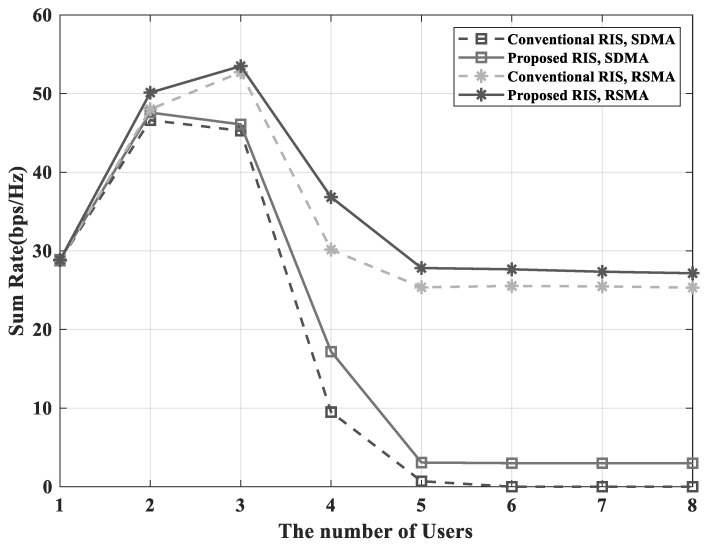
Sum-rate versus the number of users with $N_{G}=8$ and $N_{I}=16$.

Figure 7 shows the sum-rate performance according to the number of users. To apply Algorithm 1, the number of users should be less than or equal to the number of groups. The RSMA scheme had higher performance than the SDMA scheme, and the performance of the proposed RIS was also higher than the conventional RIS. When the RSMA scheme was used, the system supported eight users with an appropriate sum-rate. In contrast, the system using the SDMA scheme could support up to four users. This shows that the RSMA scheme had higher scalability than the SDMA scheme.

## 5. Conclusions

In this paper, the optimization scheme of the sum-rate for the RIS-assisted RSMA in MU-MIMO system with a reconfigurable impedance network connected by the group was proposed. First, the scattering matrix was successively optimized for each group, which was grouped by the RIS elements with the largest channel gain to the scheduled users. Then, the optimization of the RS power-splitting ratio was iteratively performed based on FP. The proposed scheme is adaptive to each UE channel and flexible in managing the interference. The simulation results showed that the proposed system had a higher sum-rate than the conventional RIS-assisted SDMA. Additionally, it can be observed that proposed system is more energy efficient and can support the users up to the number of groups with appropriate performance. Therefore, the proposed RIS-assisted RSMA scheme can be used effectively in the future B5G and 6G systems.

## Figures and Tables

**Table 1:** Simulation parameters.

Parameter	Symbol	Value
The number of transmit antennas	$N_{T}$	8
The number of UEs	$N_{K}$	3
Characteristic impedance	$Z_{0}$	50 Ω
Path-loss exponent	β	2.2
Reference path loss	$L_{0}$	30 dB
Transmit power	$P_{T}$	40 dBm
Noise power	$\sigma_{1}^{2}=\sigma_{2}^{2}=\cdots =\sigma_{N_{K}}^{2}$	−80 dBm
Initial RS power-splitting ratio	α	0.5

## Data Availability

Not applicable.

## Appendix A

When $\nu_{c}$, $\mu_{c}$, $\nu_{k}$, and $\mu_{k}$, ∀k are fixed, Equation (A1) is arranged as Equation (A2). The optimal α can be determined by setting ∂f(α)/∂α to 0, as in Equation (A3). In Equation (A3), (a) is the linear approximation at α=0.5.

(A1) $$\begin{array}{cc} \max_{\alpha } & \log_{2}\left(1+\nu_{c}\right)-\nu_{c}+2\mu_{c}\sqrt{\left(1+\nu_{c}\right)P_{c}{\left|h_{\hat{k}}\Phi Gp_{c}\right|}^{2}}-\mu_{c}^{2}P_{c}{\left|h_{\hat{k}}\Phi Gp_{c}\right|}^{2}\\ & -\sum_{k=1}^{N_{K}}\mu_{c}^{2}P_{p}{\left|h_{\hat{k}}\Phi Gp_{k}\right|}^{2}+\mu_{c}^{2}\sigma_{\hat{k}}^{2}+\sum_{k=1}^{N_{K}}\{\log_{2}\left(1+\nu_{k}\right)-\nu_{k}\\ & +2\mu_{k}\sqrt{\left(1+\nu_{k}\right)P_{p}{\left|h_{k}\Phi Gp_{k}\right|}^{2}}-\sum_{j\neq k}\mu_{k}^{2}P_{p}{\left|h_{k}\Phi Gp_{j}\right|}^{2}+\mu_{k}^{2}\sigma_{k}^{2}\} \end{array}$$

(A2) $$\begin{array}{cc} \; & \max_{\alpha }f\left(\alpha \right)\\ \; & =\max_{\alpha }2\mu_{c}\sqrt{\left(1+\nu_{c}\right)P_{c}{\left|h_{\hat{k}}\Phi Gp_{c}\right|}^{2}}-\mu_{c}^{2}P_{c}{\left|h_{\hat{k}}\Phi Gp_{c}\right|}^{2}-\sum_{k=1}^{N_{K}}\mu_{c}^{2}P_{p}{\left|h_{\hat{k}}\Phi Gp_{k}\right|}^{2}\\ & +\sum_{k=1}^{N_{K}}\left\{2\mu_{k}\sqrt{\left(1+\nu_{k}\right)P_{p}{\left|h_{k}\Phi Gp_{k}\right|}^{2}}-\sum_{j\neq k}\mu_{k}^{2}P_{p}{\left|h_{k}\Phi Gp_{j}\right|}^{2}\right\} \end{array}$$

(A3) $$\begin{array}{cc} \frac{\partial f(\alpha )}{\partial \alpha }= & -\frac{\sqrt{\left(1+\nu_{c}\right)\mu_{c}^{2}P_{T}{\left|h_{\hat{k}}\Phi Gp_{c}\right|}^{2}}}{\sqrt{1-\alpha }}+\frac{P_{T}\sum_{k=1}^{N_{K}}\mu_{k}\sqrt{\left(1+\nu_{k}\right){\left|h_{k}\Phi Gp_{k}\right|}^{2}}}{\sqrt{\alpha }}+\mu_{c}^{2}P_{T}{\left|h_{\hat{k}}\Phi Gp_{c}\right|}^{2}\\ \; & -\frac{P_{T}}{N_{K}}\sum_{k=1}^{N_{K}}\mu_{c}^{2}{\left|h_{\hat{k}}\Phi Gp_{k}\right|}^{2}-P_{T}\sum_{k=1}^{N_{K}}\sum_{j\neq k}\mu_{k}^{2}{\left|h_{k}\Phi Gp_{j}\right|}^{2}+\mu_{k}^{2}\sigma_{k}^{2}=0\\ \underset{\left(a\right)}{\approx } & -\frac{\sqrt{\left(1+\nu_{c}\right)\mu_{c}^{2}P_{T}{\left|h_{\hat{k}}\Phi Gp_{c}\right|}^{2}}}{\left(1.5-\alpha \right)/\sqrt{2}}+\frac{P_{T}\sum_{k=1}^{N_{K}}\mu_{k}\sqrt{\left(1+\nu_{k}\right){\left|h_{k}\Phi Gp_{k}\right|}^{2}}}{\left(\alpha -0.5\right)/\sqrt{2}}+\mu_{c}^{2}P_{T}{\left|h_{\hat{k}}\Phi Gp_{c}\right|}^{2}\\ \; & -\frac{P_{T}}{N_{K}}\sum_{k=1}^{N_{K}}\mu_{c}^{2}{\left|h_{\hat{k}}\Phi Gp_{k}\right|}^{2}-P_{T}\sum_{k=1}^{N_{K}}\sum_{j\neq k}\mu_{k}^{2}{\left|h_{k}\Phi Gp_{j}\right|}^{2}+\mu_{k}^{2}\sigma_{k}^{2}=0 \end{array}$$
